# Short-Term Effects of Intravitreal Ranibizumab Biosimilar Injections in Patients with Neovascular Age-Related Macular Degeneration on Retinal Nerve Fiber Layer Thickness

**DOI:** 10.3390/jcm14228225

**Published:** 2025-11-20

**Authors:** Seung Yeon Lee, Daniel Duck-Jin Hwang

**Affiliations:** 1 Department of Ophthalmology, Hangil Eye Hospital, Incheon 21388, Republic of Korea; pigglet0125@naver.com; 2 Department of Ophthalmology, Catholic Kwandong University College of Medicine, Incheon 22711, Republic of Korea

**Keywords:** neovascular age-related macular degeneration, intravitreal ranibizumab biosimilar, peripapillary retinal nerve fiber layer, optical coherence tomography

## Abstract

**Background/Objectives:** Intravitreal anti–vascular endothelial growth factor (anti-VEGF) therapy is the standard treatment for neovascular age-related macular degeneration (nAMD), but concerns remain regarding its potential effects on optic nerve structure. Evidence on the structural safety of ranibizumab biosimilars, including LucenBS^®^, is still limited. This study aimed to investigate the short-term effects of intravitreal LucenBS^®^ injections on peripapillary retinal nerve fiber layer (RNFL) thickness in previously treated nAMD patients using optical coherence tomography (OCT). **Methods:** This retrospective, observational case series included 24 eyes from 24 nAMD patients who had previously received anti-VEGF agents other than ranibizumab biosimilar. In bilateral cases, the eye that developed nAMD earlier was selected. Patients received between one and three LucenBS^®^ injections, and the mean follow-up period after the final injection was 11.92 ± 4.81 weeks. Best-corrected visual acuity (BCVA), intraocular pressure (IOP), central macular thickness (CMT), and peripapillary RNFL thickness were assessed before and after each injection using spectral-domain OCT. Sectoral and global RNFL values were compared using the Wilcoxon signed-rank test. **Results:** The mean age of participants was 74.6 ± 9.0 years, and baseline BCVA and IOP were 0.83 ± 0.66 logMAR and 14.88 ± 2.80 mmHg, respectively. RNFL thickness showed no significant changes in either global or sectoral regions after any injection (all *p* > 0.05). CMT significantly decreased after the first injection (*p* = 0.007), but remained stable with subsequent treatments. BCVA remained stable after the first and second injections, but slightly worsened after the third injection (*p* = 0.012). IOP showed no significant changes at any time point. **Conclusions:** Short-term intravitreal LucenBS^®^ injections did not induce structural alterations in the peripapillary RNFL, supporting their short-term ocular safety in previously treated nAMD patients. Although CMT improved after the first injection, functional and anatomical responses varied with repeated dosing. Larger, long-term studies are required to further validate the structural and functional safety of ranibizumab biosimilars in nAMD management.

## 1. Introduction

Neovascular age-related macular degeneration (nAMD) is a progressive retinal disorder affecting the macula and represents one of the leading causes of irreversible vision loss in the elderly population worldwide [1]. With the global rise in life expectancy, its prevalence is steadily increasing, emphasizing the need for effective therapeutic strategies to preserve visual function [2,3]. Currently, intravitreal injections of anti-vascular endothelial growth factor (anti-VEGF) agents have become the standard treatment, significantly improving the visual prognosis of patients with nAMD [4].

At present, several anti-VEGF agents are used for the treatment of nAMD, including aflibercept, brolucizumab, and ranibizumab, as well as recently introduced agents such as faricimab and conbercept [5,6,7]. Following the expiration of the original agents’ patent, several biosimilars, including Byooviz^®^, LucenBS^®^, and Enzeevu^®^, have been developed to maintain comparable therapeutic efficacy while reducing treatment-related costs [8,9,10,11]. Among these, ranibizumab is one of the most frequently prescribed in clinical practice, and LucenBS^®^ (Chong Kun Dang Pharmaceutical Corp., Seoul, Republic of Korea), a ranibizumab biosimilar, has been shown to be clinically equivalent to the reference product in terms of visual improvement, reduction in central macular thickness, and suppression of disease activity [12,13]. It was approved in Korea in 2022 for the treatment of nAMD.

Despite these therapeutic advances, concerns about the potential adverse effects of repeated intravitreal anti-VEGF injections, particularly regarding optic nerve integrity, persist and are actively under-investigated. However, data on the effects of ranibizumab biosimilars, including LucenBS^®^, on optic nerve structure are still limited. The peripapillary retinal nerve fiber layer (pRNFL), a well-established structural marker of optic nerve integrity, can be noninvasively and repeatedly assessed using optical coherence tomography (OCT) [14,15,16,17].

To address this gap, the present study evaluated pRNFL thickness using OCT after intravitreal LucenBS^®^ injections in nAMD patients with insufficient response to prior anti-VEGF therapy. The aim was to clarify the short-term effects of a ranibizumab biosimilar on the optic nerve and to explore the potential cumulative impact of repeated injections. In this study, we specifically investigated patients who had previously received anti-VEGF treatment to assess the additive or cumulative effects of additional LucenBS^®^ injections.

## 2. Materials and Methods

The study protocol was reviewed and approved by the Institutional Review Board of Hangil Eye Hospital (IRB No. 23006) and adhered to the principles outlined in the Declaration of Helsinki. Given its retrospective design, the requirement for written informed consent was waived by the ethics committee. We used EndNote Version X9.3.1. for reference management and ChatGPT (OpenAI) Version 5.1 for language refinement and proofreading support.

### 2.1. Participants

This was a retrospective, observational, and consecutive case series study. Patients who received intravitreal injections of a ranibizumab biosimilar (LucenBS^®^, Chong Kun Dang Pharmaceutical Corp., Seoul, Republic of Korea) at Hangil Eye Hospital (Incheon, Republic of Korea) between May 2024 and June 2025 were included. A total of 24 eyes of 24 patients with nAMD were enrolled, including 13 men and 11 women. All patients were non-naïve, previously received anti-VEGF treatments other than the ranibizumab biosimilar. In patients who received bilateral injections, the eye initially diagnosed with nAMD was included for analysis. Eyes with ocular conditions that could affect RNFL thickness—such as retinal vein occlusion, uveitis, glaucoma, optic nerve diseases, or central serous chorioretinopathy—were excluded. Eyes with a prior history of ocular trauma or any previous interventions that could influence RNFL thickness—such as vitrectomy or intraocular laser treatment—were excluded.

All intravitreal injections were performed by a single ophthalmologist (D.D.H). Polypoidal choroidal vasculopathy (PCV) was identified by the presence of polypoidal lesions with or without associated branching vascular networks, whereas eyes exhibiting retinal–retinal or retinal–choroidal anastomoses were classified as retinal angiomatous proliferation (RAP). Cases that did not meet the diagnostic criteria for PCV or RAP were categorized as typical nAMD with type 1 or type 2 choroidal neovascularization. Baseline and follow-up data, including spectral domain OCT (SD-OCT, Heidelberg Engineering, Heidelberg, Germany) and medical records, were retrospectively analyzed at baseline and at the earliest follow-up after the first, second, and third intravitreal LucenBS^®^ injections.

### 2.2. Ophthalmic Examinations

Best-corrected visual acuity and intraocular pressure were assessed using slit-lamp biomicroscopy, fundus photography, and SD-OCT at baseline (prior to the first LucenBS^®^ injection) and after each injection (first, second, and third). Peripapillary RNFL thickness was measured using the SD-OCT program (Spectralis Nsite Axonal Analytics Software Version 6.9a, Heidelberg Engineering, Heidelberg, Germany). The peripapillary RNFL was segmented into six regions according to the clock-hour distribution around the optic disk: temporal (315–45°), superotemporal (45–90°), superonasal (90–135°), nasal (135–225°), inferonasal (225–270°), and inferotemporal (270–315°) sectors. The global RNFL thickness was defined as the average value across all six sectors. Scans with poor segmentation quality were excluded from the analysis. Only scans with an automatic real-time (ART) score of ≥16 and a signal-to-noise ratio ≥ 15 dB were included. Central macular thickness was determined as the average thickness of the central 1 mm diameter circle using the software provided with the SD-OCT device. Macular thickness measurements were obtained using a 30° volume scan centered on the fovea with central fixation assistance and an inter-scan distance of 250 μm.

### 2.3. Statistical Analysis

Statistical analyses were performed IBM SPSS Statistics for Windows, Version 25.0 (IBM Corp., Armonk, NY, USA). Data are presented as mean ± standard deviation. The Wilcoxon signed-rank test was used to evaluate the statistical significance of changes from baseline to each follow-up time point. A *p*-value of <0.05 was considered to be statistically significant.

## 3. Results

The baseline characteristics of the 24 patients are summarized in Table 1. Among them, 13 were male (54.2%), and the mean age at the time of the first ranibizumab biosimilar treatment was 74.62 ± 8.99 years. Among the subtypes of choroidal neovascularization (CNV), the typical form was the most prevalent, observed in twenty-one eyes (87.5%), followed by polypoidal choroidal vasculopathy (PCV) in two eyes (8.3%) and retinal angiomatous proliferation (RAP) in one eye (4.2%). Of the treated eyes, eight were right eyes (33.3%) and sixteen were left eyes (66.7%). For each patient, the study eye was selected as the one diagnosed with nAMD that had previously received other anti-VEGF agents and was subsequently switched to LucenBS^®^ therapy. In cases where both eyes met these criteria, the eye diagnosed earlier and received a greater number of LucenBS^®^ injections was selected. A total of 18 patients (75.0%) had systemic comorbidities such as hypertension and diabetes; specifically, 16 patients (66.7%) had hypertension, and 8 (33.3%) had diabetes. Prior to enrollment, all patients had received anti-VEGF therapy excluding the ranibizumab biosimilar, with a mean number of 14.67 injections (range, 3–74). The mean baseline best-corrected visual acuity (BCVA, logMAR) was 0.83 ± 0.66, and the mean spherical equivalent refractive error (SE) was −0.12 ± 1.06 diopters. The mean intraocular pressure (IOP) at baseline was 14.88 ± 2.80 mmHg. The mean follow-up period after last injection was 11.92 ± 4.81 weeks (range, 5–26). The mean follow-up durations after each injection were 79.33 ± 34.13 days for the first injection (*n* = 24), 96.93 ± 34.73 days for the second injection (*n* = 15), and 77.89 ± 25.76 days for the third injection (*n* = 9).

### 3.1. Changes in the BCVA and IOP

The mean best-corrected visual acuity (BCVA), expressed as the logarithm of the minimum angle of resolution (log MAR) at baseline, was 0.83 ± 0.66 (range 0.00–2.00). No significant changes were observed after the first and second injections: 0.81 ± 0.65 after the first injection (*p* = 0.959, Wilcoxon signed-rank test) and 0.82 ± 0.70 after the second injection (*p* = 0.172, Wilcoxon signed-rank test). However, BCVA significantly worsened after the third injection, reaching 0.88 ± 0.55. (*p* = 0.012, Wilcoxon signed-rank test)

The mean intraocular pressure (IOP) was 14.88 ± 2.80 mmHg at baseline, 15.83 ± 3.14 mmHg after the first injection, 15.40 ± 2.03 mmHg after the second injection, and 15.40 ± 2.03 mmHg after the third injection. There were no statistically significant changes in IOP across all time points (all *p* > 0.05, Wilcoxon signed-rank test).

### 3.2. Changes in the Peripapillary RNFL Thickness

**Figure 1:**
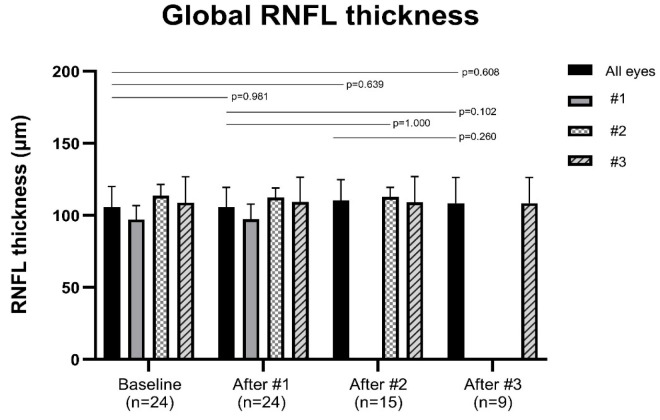
Changes in peripapillary retinal nerve fiber layer (RNFL) thickness in the global sector. The peripapillary RNFL was divided into six sectors around the optic disk: temporal, superior temporal, superior nasal, nasal, inferior nasal, and inferior temporal. The global RNFL thickness was defined as the average value across all six sectors. Patients were grouped according to the number of injections, and the graphs are stratified by injection number. Data are presented as mean and standard deviation. *p*-values were calculated for pairwise comparisons between baseline and each post-injection time point (after the first, second, and third injections), as well as between injection time points. No statistically significant changes in RNFL thickness were observed in any sector. *p*-values were obtained using the Wilcoxon signed-rank test.

**Figure 2:**
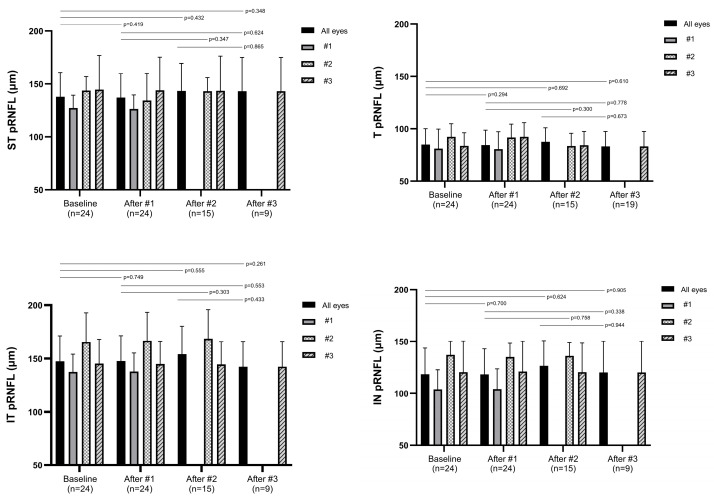

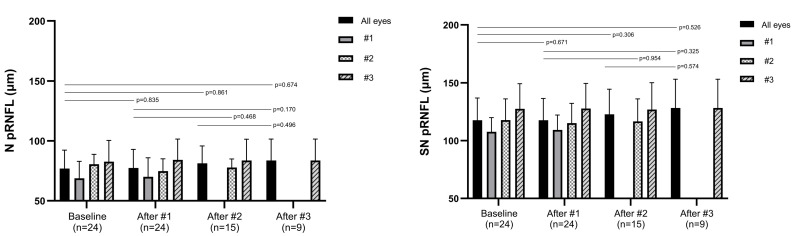
Changes in peripapillary retinal nerve fiber layer (RNFL) thickness in six sectors—temporal (T), superior temporal (ST), superior nasal (SN), nasal (N), inferior nasal (IN), and inferior temporal (IT)— following intravitreal ranibizumab biosimilar injections. Patients were grouped according to the number of injections, and the graphs are stratified by injection number. Data are presented as mean and standard deviation. *p*-values were calculated for pairwise comparisons between baseline and each post-injection time point (after the first, second, and third injections), as well as between injection time points. No statistically significant changes in RNFL thickness were observed in any sector. *p*-values were obtained using the Wilcoxon signed-rank test.

Figure 1 summarizes the changes in peripapillary retinal nerve fiber layer (RNFL) thickness in the global (G) sector. Figure 2 summarizes the changes in peripapillary RNFL thickness in the temporal (T), superior temporal (ST), superior nasal (SN), nasal (N), inferior nasal (IN), and inferior temporal (IT) sectors. Table 2 presents the mean RNFL thickness values (±SD) at each injection time point, organized by injection number (columns) and sector (rows). The *p*-values for comparisons with baseline are shown in parentheses. There were no significant changes in RNFL thickness in any sector after each injection compared to baseline. In addition, no significant differences were observed among the post-injection time points (all *p* > 0.05, Wilcoxon signed-rank test).

### 3.3. Changes in the Central Macular Thickness

The baseline central macular thickness (CMT) was 371.46 ± 276.47 μm. It significantly decreased to 336.25 ± 212.96 μm after the first injection (*p* = 0.007, Wilcoxon signed-rank test). However, no further significant changes in CMT were observed with subsequent injections: 374.73 ± 276.03 μm after second injection (*p* = 0.530, Wilcoxon signed-rank test), and 468.11 ± 323.96 μm after third injection (*p* = 0.906, Wilcoxon signed-rank test).

## 4. Discussion

This is the first study to investigate the short-term effects of intravitreal LucenBS^®^ injections, a ranibizumab biosimilar developed in Korea, on the peripapillary retinal nerve fiber layer. This study focused on patients with neovascular age-related macular degeneration who had shown insufficient response to prior anti-VEGF therapy. Across follow-up visits after one to three injections, no significant changes in RNFL thickness were observed in any group.

Ranibizumab is a monoclonal antibody fragment targeting VEGF-A that effectively inhibits abnormal angiogenesis and increased vascular permeability through its low molecular weight and high binding affinity [18,19]. LucenBS^®^, a ranibizumab biosimilar developed in Korea, provides equivalent efficacy and safety to the reference drug at a lower cost, offering a practical alternative for nAMD patients requiring long-term, repeated treatment [12,13]. A phase 3 randomized clinical trial confirmed that LucenBS^®^ achieved visual and anatomical outcomes comparable to those of reference ranibizumab in treatment-naïve nAMD patients [12]. Moreover, a real-world switching study demonstrated favorable short-term anatomical responses in patients who transitioned from bevaizumab to LucenBS^®^ [20].

However, treatment response may differ in previously treated non-naïve eyes. In a Japanese cohort with myopic CNV, ranibizumab biosimilar therapy produced significant anatomical improvement in both treatment-naïve and previously treated eyes, but functional recovery such as BCVA was significantly limited to naïve cases [21]. These findings suggest that, while ranibizumab biosimilars demonstrate therapeutic equivalence overall, their effects may vary depending on prior anti-VEGF exposure. Although the potential impact of repeated anti-VEGF injections on optic nerve structure is an important concern in chronic management, no prior studies have examined RNFL thickness changes following LucenBS^®^ injections, and evidence regarding structural safety to non-naïve patents remains limited.

Previous reports have presented conflicting evidence regarding the effect of intravitreal anti-VEGF injections on RNFL. Martínez-de-la-Casa et al. reported RNFL thinning after 12 months of ranibizumab therapy [22], and a subsequent long-term follow-up study extending to 8 years confirmed significant RNFL thinning [23]. Wang et al. further suggested that the degree of thinning increased with the cumulative number of injections [24]. A proposed mechanism is that VEGF acts as a neuroprotective factor for retinal ganglion cell survival and neuronal nutrition, and repeated VEGF inhibition may impair this function, resulting in RNFL loss [25,26,27]. Another hypothesis is that acute intraocular pressure spikes caused by sudden intravitreal volume changes immediately after injection may compromise optic nerve perfusion and induce mechanical injury [28,29]. Wen et al. reported reduced optic nerve head blood flow associated with acute IOP elevation after anti-VEGF injections [30], while Soheilian et al. demonstrated that anterior chamber paracentesis, performed to mitigate acute IOP elevation, could prevent RNFL thinning [31].

In contrast, a larger body of evidence has shown that anti-VEGF injections do not significantly affect RNFL thickness. Shin et al., in a meta-analysis, found no relationship between repeated injections or post-injection IOP fluctuations and RNFL changes [32]. Ahn et al. also reported no meaningful change in RNFL thickness between eyes treated with ranibizumab or aflibercept and their untreated fellow eyes after 12 months in treatment-naïve nAMD patients [33]. Wang et al. noted that, although peripapillary vessel density may transiently decrease after anti-VEGF treatment, the association with RNFL loss was inconsistent, and any structural changes generally recovered within months. Furthermore, they showed that transient post-injection IOP elevations did not reach a level sufficient to cause structural damage and that long-term IOP remained stable [34]. Swaminathan et al. also demonstrated that, even in nAMD patients with coexisting glaucoma who are relatively more vulnerable to IOP fluctuations, eyes receiving anti-VEGF injections did not exhibit greater RNFL loss compared with untreated eyes, suggesting that acute IOP fluctuations are unlikely to result in permanent structural damage [35].

Overall, recent evidence increasingly supports the view that anti-VEGF injections do not have a significant effect on RNFL thickness [32,33,34,35,36,37]. Reports of RNFL thinning in earlier studies may be explained by their reliance on global RNFL measurements without quadrant-or sector-based analysis [22,24,37,38]. This approach could have led to localized changes from other causes being misinterpreted as overall RNFL decline and limited the ability to identify the exact origin and distribution of the changes. In addition, most of these studies were based on follow-up periods longer than one year, raising the possibility that the observed changes reflected the natural progression of the disease or aging process [29,34,39] rather than a drug-related adverse effect.

Our findings are consistent with prior reports showing no RNFL alterations after anti-VEGF injections, and further demonstrate that LucenBS^®^ does not exert short-term deleterious effects on optic nerve structure across various nAMD subtypes. Long-term IOP remained stable, and no complications associated with acute IOP elevation were observed. The absence of structural changes, even after three repeated injections, alleviates concerns about optic nerve toxicity and supports the clinical safety of LucenBS^®^ use.

In terms of visual outcomes, BCVA did not change after one or two injections, but showed a trend toward decline after the third injection. Spooner et al., in a large meta-analysis, reported that, while anti-VEGF therapy improves vision initially, the benefit diminishes over time, with mean vision falling below baseline by approximately 8.11 letters after two years [40]. Similarly, long-term studies such as the CATT 5-year and SEVEN-UP trials also demonstrated gradual vision loss over time [41,42]. In our cohort, patients were not treatment-naïve and had prior exposure to other anti-VEGF agents, and the mean follow-up period extended beyond 10 weeks. Notably, among the nine patients who received three injections, eight showed a decline in BCVA, all of whom had typical AMD subtype. At the follow-up visit after the third injection, these eyes exhibited persistent or recurrent disease activity and subsequently required additional anti-VEGF treatment. This pattern suggests that the BCVA decline in these cases was driven by ongoing disease activity, rather than a reduction in the pharmacologic efficacy of LucenBS^®^. However, given the small sample size and the inclusion of non-naïve patients, identifying definitive contributing factors remains challenging. Further studies with larger cohorts will be needed to clarify the predictors of visual decline in patients receiving ranibizumab biosimilars. CMT decreased after the first injection compared to baseline, consistent with the findings of Bae et al., who reported anatomical stabilization and the suppression of exudation following brolucizumab in non-naïve nAMD patients [43]. However, in some cases, CMT showed a tendency to increase again after repeated injections, highlighting the need for long-term follow-up studies to evaluate the durability of treatment effects.

This study has several limitations. First, as a retrospective single-center study, the sample size was small, and follow-up intervals were not uniform. In some patients, more than three months elapsed between injections and follow-up visits, limiting the precise quantification of direct treatment effects. Second, the maximum number of injections was limited to three, restricting generalization to long-term repeated use. Third, the absence of a control group, such as untreated fellow eyes or eyes treated with the original ranibizumab, which may weaken casual interpretation of the findings. Finally, since this study included patients previously treated with anti-VEGF agents, the pure effects of LucenBS^®^ could not be isolated. Prospective studies in treatment- naïve patients will be necessary to address this issue.

Nevertheless, this study provides the first evidence that repeated intravitreal ranibizumab biosimilar (LucenBS^®^) injections in previously treated nAMD patients do not induce structural changes in the peripapillary RNFL. Furthermore, the results may provide evidence for ranibizumab biosimilar as a safe short-term therapeutic alternative. Future multi-center prospective studies with longer follow-up should be expanded to further establish the structural and functional safety of long-term ranibizumab biosimilar therapy.

## Figures and Tables

**Table 1:** Baseline characteristics of patients who received ranibizumab biosimilar injections.

Characteristics	Value
No. of patients	24
Sex, male–female	13:11
Age of the first ranibizumab biosimilar injection (years)	74.62 ± 8.99 (range, 55–90)
Type of AMD (*n* %), PCV–typical AMD–RAP	2 (8.3):21 (87.5):1 (4.2)
Laterality, OD:OS	8:16
Systemic disease	
Hypertension (*n*)	16
Diabetes (*n*)	8
No. of previous anti-VEGF injections	14.67 ± 15.40 (range, 3–74)
Corrective visual acuity (logMAR)	0.83 ± 0.66 (range, 0.00–2.00)
Refractive error (SE)	−0.12 ± 1.06 (range, −2.875–+1.50)
IOP (mmHg)	14.88 ± 2.80 (range, 9–20)

Values are presented as number (%) or mean ± standard deviation. AMD = age-related macular degeneration; PCV = polypoidal choroidal vasculopathy; RAP = retinal angiomatous proliferation; OD = right eye; OS = left eye; VEGF = vascular endothelial growth factor; logMAR = logarithm of the minimum angle of resolution; SE = spherical equivalent; IOP = intraocular pressure.

**Table 2:** Changes in peripapillary retinal nerve fiber layer thickness (μm) across sectors following intravitreal ranibizumab biosimilar treatment.

	Baseline	1st Injection	2nd Injection	3rd Injection
Global	105.58 ± 14.34	105.58 ± 13.91	110.47 ± 14.28	108.44 ± 17.76
		(0.981)	(0.639)	(0.608)
Temporal	84.88 ± 15.15	84.46 ± 14.27	87.53 ± 13.40	83.22 ± 14.21
		(0.294)	(0.692)	(0.610)
Superior temporal	137.92 ± 22.82	137.13 ± 22.54	143.40 ± 25.86	143.22 ± 31.78
		(0.419)	(0.432)	(0.348)
Superior nasal	117.67 ± 19.21	117.71 ± 18.88	122.80 ± 21.78	128.33 ± 24.88
		(0.671)	(0.306)	(0.526)
Nasal	76.92 ± 15.37	77.42 ± 15.57	81.33 ± 14.44	83.67 ± 17.94
		(0.835)	(0.861)	(0.674)
Inferior nasal	118.42 ± 25.39	118.29 ± 24.82	126.73 ± 23.98	120.22 ± 29.97
		(0.700)	(0.624)	(0.905)
Inferior temporal	147.38 ± 23.77	147.63 ± 23.56	154.13 ± 25.91	142.33 ± 23.51
		(0.749)	(0.555)	(0.261)

Values are presented as mean ± standard deviation. The *p*-values for comparisons between baseline and each injection group are shown in parentheses (Wilcoxon signed-rank test).

## Data Availability

On reasonable request, the corresponding author will provide the datasets created and analyzed during the current work.

## Abbreviations

The following abbreviations are used in this manuscript:

RNFL	Retinal nerve fiber layer
pRNFL	Peripapillary retinal nerve fiber layer
nAMD	Neovascular age-related macular degeneration
OCT	Optical coherence tomography
VEGF	Vascular endothelial growth factor
BCVA	Best-corrected visual acuity
IOP	Intraocular pressure
CMT	Central macular thickness
log MAR	Logarithm of the minimum angle of resolution
SE	Spherical equivalent
CNV	Choroidal neovascularization
PCV	Polypoidal choroidal vasculopathy
RAP	Retinal angiomatous proliferation
ART	Automatic real-time
